# Viral-Vector-Delivered Anti-Angiogenic Therapies to the Eye

**DOI:** 10.3390/pharmaceutics13020219

**Published:** 2021-02-05

**Authors:** Sanna Koponen, Emmi Kokki, Kati Kinnunen, Seppo Ylä-Herttuala

**Affiliations:** 1 A.I. Virtanen Institute for Molecular Sciences, University of Eastern Finland, P.O. Box 1627, 70211 Kuopio, Finland; sanna.k.koponen@uef.fi (S.K.); Emmi.kokki@uef.fi (E.K.); 2 Department of Ophthalmology, Kuopio University Hospital, 70211 Kuopio, Finland; Kati.Kinnunen@kuh.fi; 3 Gene Therapy Unit, Kuopio University Hospital, 70211 Kuopio, Finland

**Keywords:** gene therapy, ocular neovascularization, viral vectors, preclinical, clinical trials, anti-angiogenesis, adeno-associated virus

## Abstract

Pathological vessel growth harms vision and may finally lead to vision loss. Anti-angiogenic gene therapy with viral vectors for ocular neovascularization has shown great promise in preclinical studies. Most of the studies have been conducted with different adeno-associated serotype vectors. In addition, adeno- and lentivirus vectors have been used. Therapy has been targeted towards blocking vascular endothelial growth factors or other pro-angiogenic factors. Clinical trials of intraocular gene therapy for neovascularization have shown the treatment to be safe without severe adverse events or systemic effects. Nevertheless, clinical studies have not proceeded further than Phase 2 trials.

## 1. Introduction

Ocular neovascularization is one of the most common causes of moderate and severe vision loss in developed countries. Pathological angiogenesis, which is mainly induced through vascular endothelial growth factors (VEGF) and their receptors, is involved in many of these vision-impairing ocular disorders, such as age-related macular degeneration (AMD), diabetic retinopathy (DR), and corneal neovascularization [1]. In the healthy eye, VEGF is necessary for the maintenance of retinal pigment epithelium (RPE) and choriocapillaris [2]. As the treatment of the eye possesses several challenges, oral or topical administration of the drug does not achieve therapeutic concentration in the eye [3]. As most of the vision-impairing diseases affect the retina, repeated anti-VEGF injections into the vitreous are currently the most commonly used treatment method in order to inhibit angiogenesis [4]. The treatment with anti-VEGF injections is effective, but it also has disadvantages due to the need for repeated injections, which places a significant burden on the patients and healthcare system and poses a risk of adverse events including inflammation, retinal detachment, and subretinal and vitreous hemorrhage.

Gene therapy with viral vectors for ocular diseases has shown great promise for the future. It provides an alternative treatment for re-occurring injections. Viral vectors are efficient in transducing different tissues and provide long-term treatment options [5]. Successful gene therapy relies on efficient gene delivery to the target cells, which can be achieved by selecting the right delivery system, specific promoter elements, and administration route. An eye is an optimal gene therapy target as it can be easily injected by using only small injection volumes of the virus, still leading to successful expression inside the eye [6,7]. In addition, due to the blood–retina barrier, the gene therapy can be targeted with high titers into the eye, minimizing systemic biodistribution and side effects. Genome editing has brought new possibilities to edit gene expression in the eye. Food and Drug Administration (FDA) approved the first gene transfer treatment for the ocular disease in 2017, where a subretinal delivery of adeno-associated virus serotype 2 (AAV2)-mediated voretigene neparvovecrzyl (Luxturna^®^,Spark Therapeutics, Inc., Philadephia, PA, USA) is administered for treating inherited biallelic RPE65 mutation-associated retinal dystrophy [8]. The approval of the gene therapy proves that it is a usable tool for treating ocular diseases.

## 2. Angiogenesis

Angiogenesis is the growth of blood vessels and is essential for organ growth in the embryo and in the placenta during pregnancy, in the female reproductive system during ovulation and menstruation and wound healing in adults [9]. Angiogenesis is tightly controlled and balanced between proangiogenic and anti-angiogenic factors. One of the most important proangiogenic factors is the VEGF, which induces endothelial cell proliferation, migration, and new vessel formation. The VEGF signals are mediated through tyrosine kinase receptors VEGFR-1, -2, and -3 and co-receptors neuropilins 1 and 2 [10]. VEGF-A is one of the strongest vascular permeability inducers, which expression is increased in hypoxic conditions. Soluble VEGF receptors sVEGFR-1, -2, and -3 function as competitive inhibitors of the binding of VEGFs to the membrane-bound receptors, therefore regulating VEGF signaling [11]. In addition there are other molecules that affect angiogenesis, such as angiopoietin-2 (ANG-2), fibroblast growth factors (FGF), and chemokines, released by hypoxic, inflammatory, or tumor cells [12].

**Figure 1:**
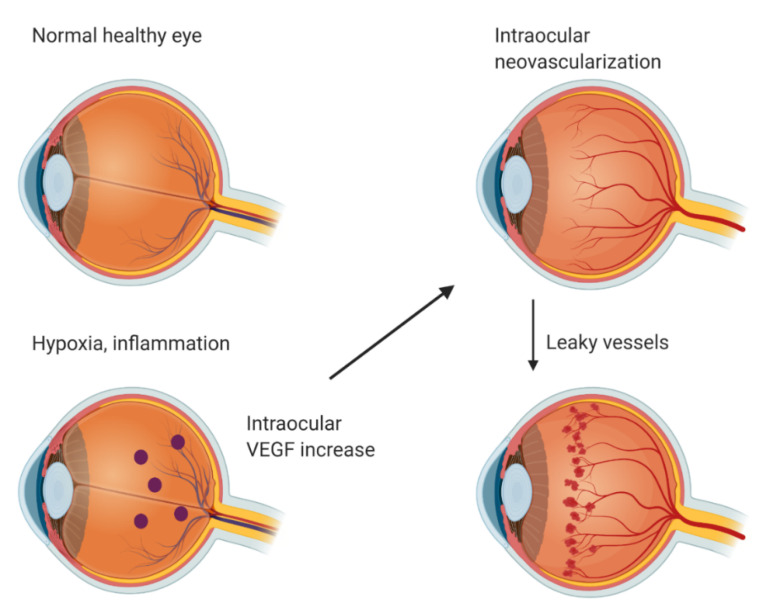
Intraocular neovascularization. Hypoxia or inflammation upregulates VEGF expression, which leads to intraocular neovascularization and, ultimately, leaky vessels. Created with BioRender.com.

In a normal healthy eye, VEGF is expressed in the retina, choroid-RPE, iris, and conjunctiva [2]. In the retina, VEGF expression is localized in the ganglion, inner nuclear, and RPE cell layers. VEGF is crucial for cellular homeostasis as a neurotrophic and cell survival factor but it is also one of the critical mediators of pathological neovascularization [13]. Intraocular VEGF is upregulated by hypoxia and inflammation (Figure 1). Upregulated VEGF drives neovascularization and increases vascular permeability. This leads to different complications such as leaky vessels, retinal detachment, fibrovascular proliferation, retinal exudation, edema, and ultimately photoreceptor neuron death and blindness. Pathological angiogenesis in the eye includes many diseases—for example, AMD, DR, retinopathy of prematurity (ROP), and corneal neovascularization [1].

Currently available treatment options for ocular neovascularization, especially for wet AMD, include photodynamic therapy (PDT), laser therapy, and anti-VEGF therapies [14]. The use of laser therapy and photodynamic therapy for treatment has declined over time [15]. Laser surgery and PDT have a limited visual improvement potential and, in addition, panretinal photocoagulation laser treatment for DR even has a risk of producing vision loss due to its inherently destructive approach [16,17], although when using newer laser technologies, the risk is lower [18]. The three most commonly used anti-VEGF therapies are aflibercept, ranibizumab, and bevacizumab, which bind to VEGF and prevent their signal transduction through VEGFR [4]. These VEGF inhibitors are injected intravitreally into the eye. Aflibercept is a recombinant fusion protein of human VEGFR-1 and VEGFR-2 binding domains fused with the Fc domain of human IgG1 [19]. Ranibizumab is a recombinant antibody fragment of the humanized anti-VEGF monoclonal antibody. Bevacizumab is a full-length recombinant humanized anti-VEGF monoclonal antibody (IgG). The FDA and European Medicines Agency (EMA) have approved aflibercept and ranibizumab for the treatment of AMD. Bevacizumab has not been approved by the FDA or EMA [20] for ophthalmic use, but its safety and efficacy have been shown in multiple clinical AMD and DR trials. Anti-VEGF therapies have been effective to treat ocular neovascularization [4]. However, intravitreal injections are required monthly, which places a significant burden on the patients and the healthcare system. Injections also pose a risk of serious ocular adverse events, including endophthalmitis, retinal detachment, and subretinal and vitreous hemorrhage.

## 3. Routes of Administration for Ocular Gene Therapy

The ocular tissues can be reached by either local or systemic administration [3]. The eye has many tissue barriers that limit the access of drugs to theirs targets, such as the corneal– and conjunctival–epithelial, blood–aqueous, and blood–retinal barriers. In particular, the blood–retinal barrier limits drug diffusion from systemic circulation. Systemic administration has side effects such as systemic toxicity. Topical drug administration is non-invasive and easy to apply. It is used for the treatment of anterior segment disorders. However, eye drops are ineffective at reaching posterior tissues and have poor ocular bioavailability. Periocular, intracameral, intravitreal, and subretinal administrations are invasive methods [7]. Periocular administration happens via peribulbar, retrobulbar, posterior juxtascleral, subtenon, and subconjunctival injections. The subconjunctival injection is administered between the bulbar conjunctiva and sclera and is a minimally invasive way to target the delivery of drugs to the choroid and retina [21]. Subconjunctival injection of AAV vectors has been shown to transduce the eyelid, conjunctiva, cornea, optic nerve, and periocular tissues including muscle [22]. However, the injected drug might absorb into the lymphatic and blood circulation, which limits its ocular bioavailability [23]. The intracameral injection is administered into the anterior chamber [24]. Intracameral injection of lentivirus vector has been shown to transduce trabecular meshwork and corneal endothelial cells. AAV2, AAV3, and AAV4 cannot efficiently transduce TM [25]. Synthetically developed AAV, Anc80L65, has been shown to transduce the anterior segment, including the TM, the corneal stroma, and endothelial cells. Self-complementary AAV2, scAAV5, and scAAV8 have been shown to transduce the ciliary body, the iris, and the corneal endothelium of ocular hypertensive eyes [26]. The intracameral injection might be an inefficient method because of the rapid turnover of aqueous humor and the short contact time with ocular tissues [21]. The suprachoroidal injection is administered below the sclera and above the choroid. It is minimally invasive and a simple way to target drugs to the posterior segment of the eye. Suprachoroidal AAV8 vector injection was shown to transduce the retina and RPE [27]. Injection into suprachoroidal space does not interfere in the optical pathways; however, macromolecules are cleared rapidly [7].

In preclinical trials, the subretinal injections are delivered by the transcorneal route through the pupil passing the lens and retina, by the trans-scleral route through the vitreous, and by the trans-scleral route through the choroid and Bruch’s membrane [28]. Meanwhile, for humans, subretinal injection can use the pars plana vitrectomy approach, in which the core vitreous is removed [29,30]. A needle is guided into the subretinal space and a bleb is formed by a small infusion of balanced salt solution. For gene delivery, the same injection channel through the neural retina is used to inject the therapeutic agent into the subretinal space. Compared with direct delivery into the subretinal space, this approach offers better access to the correct plane as well as minimizing vector loss but also exposes patients to surgical complications. Subretinal AAV2 and AAV5 injection have been shown to transduce photoreceptor and RPE cells in the murine retina [31]. The subretinal injection is administered into the subretinal space and might cause ocular damage such as lesions, hemorrhages, fibrosis, and retinal detachment [7]. The subretinal AAV transduction profile of photoreceptors differs significantly between neonatal and adult mouse retinas [32]. Photoreceptor development and integrity play a major role in the efficacy of subretinal AAV-mediated transduction. It was shown that rod outer segments are critical modulators of efficient rod transduction, which increases during mouse postnatal retinal development. The loss of rod outer segments in diseased retinas affected the efficacy of gene transfer by limiting the ability of AAVs to infect dying rods efficiently. The altered development of rod outer segments was associated with an increase in the transduced number of cones and a strong reduction in rod transduction. To achieve the best transduction efficiency in photoreceptor cells, the timing of the treatment is to be considered.

Intravitreal injection is administered into the vitreous [7]. Compared with subretinal injection, administration to the vitreous is relatively easy, and high doses are possible. The intravitreal injection route is directly applicable to patients and it is routinely used to administer ophthalmic drugs. As subretinal injections need to be performed in an operating theater, intravitreal injections are less invasive and less expensive [29]. However, it might have adverse effects such as retinal detachment, endophthalmitis, and an increase in intraocular pressure [33]. Compared with the other intraocular injections, the intravitreal approach may be a more efficient route for therapeutic gene delivery as it can potentially expose the entire retina [33], although it mostly targets cells in the inner retina [34,35,36] and anterior chamber [37,38,39]. It is important to consider which delivery route to choose to achieve efficient gene expression where it is needed.

## 4. Gene Therapy and Viral Vectors

Gene therapy involves the transfer of nucleic acids into a cell to either correct dysfunctional gene or to provide new cellular functions [40]. Gene therapy can be achieved by the substitution of the altered gene, inhibiting the gene expression or insertion of a new gene [41]. Genes can be transferred by viral or non-viral vectors. Non-viral gene delivery can be achieved by physical or chemical methods. In the physical methods, the permeability of the cell membrane is increased by physical force, and in the chemical methods, natural or synthetic carriers are used. Compared with viral vectors, non-viral vectors are less immunogenic, less toxic, and easily produced. On the other hand, they are less effective at expressing transgenes at the therapeutic level. Viral vectors are more efficient in transducing cells and at inducing long-term gene expression than non-viral vectors [42]. Gene expression time is short with non-viral vectors versus viral vectors, where gene expression can last for weeks, months, or even some years. Viral vectors have limitations—for example, limited transgene capacity or they might cause immune responses. There are many different viral vectors used in gene therapy—for example, adeno-, adeno-associated-, retro-, lenti-, baculo-, herpes simplex-, and Epstein–Barr viruses. Successful gene therapy relies on efficient gene delivery to target cells and long-term gene expression [7]. It can be achieved by selecting the appropriate delivery system, specific promoter elements, and administration route. As a target for gene therapy, the eye has many advantages: eye anatomy is well known, it is easy to access and examine, it is relatively immune-privileged, and study-wise, the other eye can be used as a control.

Adenovirus (Adv) is a non-enveloped double-stranded DNA vector, which can transduce dividing and non-dividing cells [7,43]. Adenovirus can carry large genes of approximately 35 kb [44] and it does not integrate into the host cell genome. There are over 50 different human adenovirus serotypes. Adenoviral vectors transduce different types of cells in the eye [39]. After the intravitreal injection of the adenoviral vector expressing green fluorescent protein (GFP) transduced corneal endothelial cells, trabecular meshwork and iris cells. In subretinally injected eyes, transduction was detected in photoreceptors and RPE cells. In one study, adenoviral-vector-expressing β-galactosidase gene (LacZ) was detected in the nerve cell layer and ganglion cell layer of the retina [45]. In another, adenovirus serotype 5 was shown to transduce efficiently the anterior chamber corneal endothelium, trabecular meshwork, ciliary body, iris and some Müller, and inner nuclear layer (INL) cells [6]. The highest transduction was observed after 7 days of injection. After 1 month of injection, GFP expression was detected mainly in RPE cells. An inflammatory response was observed after 3 and 7 days of injection by an increasing number of F4/80-immunoreactive cells. The time range of how long the transgene is expressed also depends on the Adv serotype [39,46]. It was shown that the Ad35 vector had prolonged transgene expression compared with the Ad5 vector. Transgene expression was detected up to 6 months post Ad35 injection [39] but diminished by 8 months [46]. This is significantly longer than the gene expression seen in animal models after the most often used Ad5 gene delivery. The expression after intravitreal and subretinal Ad5-mediated injection is a couple of weeks [39,46,47]. Adenoviral gene therapy in clinical trials has shown a mild inflammatory response which has been easily managed [48,49].

Adeno-associated virus (AAV) is a single-stranded DNA vector, which can transduce dividing and non-dividing cells [50]. AAV provides long-term gene expression ranging from months to years in preclinical trials [51] and it can integrate into the host cell genome. There is a concerning possibility that integration of the AAV vector might cause insertional mutagenesis, even though the majority of experiments have shown that AAV vectors are safe and potential genotoxic effects can be minimized by vector design [50]. In addition, 3-year follow-up showed detained improvement in best corrected visual acuity (BCVA) after subretinal AAV2-mediated gene therapy in patients with Leber congenital amaurosis type 2 [52]. AAV has many different serotypes, which transduce different cells in the eye. AAV2-expressing GFP has been shown to transduce RPE, the retinal ganglion cell layer (RGCL), and some cells in the INL and the outer plexiform layer (OPL) [6]. After 1 month, GFP expression was also detected in the optic nerve, inner plexiform layer, ciliary body, trabecular meshwork, and iris. After 3 and 6 months of injection, expression was detected in RGCL and INL. In one study, subretinal injection of AAV5 and AAV2 was shown to transduce photoreceptor and RPE cells in the murine retina [31]. AAV5 transduced more photoreceptor cells than AAV2 and expression levels were maintained for up to 31 weeks, which was the latest point tested. The time course for expression was faster with AAV5 than with AAV2. Only minimal to moderate ocular inflammatory responses have been detected after AAV injections into the eye in animal models [53,54] and clinical trials [53,55,56,57,58].

Lentiviruses (LV) and retroviruses are single-stranded RNA vectors [59]. Lentiviruses can transduce dividing and non-dividing cells but retroviruses can transduce only dividing cells. Both virus vectors can integrate stably into the host cell genome. The most commonly used retro- and lentiviral vectors for gene therapy are the human immunodeficiency virus type 1 (HIV-1) and murine Moloney leukemia virus (MLV) [60]. The use of retroviruses for gene therapy has been reduced [7] after a study outcome where four patients developed T cell leukemia 31–68 months after the gamma retroviral gene delivery [61]. As a consequence of the vector integration, proto-oncogenes were activated in all four patients. Lentivirus vectors transduce different cells in the eye. It was shown that lentivirus vector expressing GFP transduce RPE, corneal endothelium, trabecular meshwork, ciliary body, iris pigmented epithelium, anterior chamber, INL, and ocular muscles after intravitreal injection [6]. It was shown that subretinal injection of lentiviral vector expressing GFP transduced RPE [62], choroid, and sclera layers [63]. Inflammatory responses in eyes treated with lentiviral vector injections have been shown to be very moderate or not significant [64,65]. In one study, subretinal lentiviral vectors have shown strong retinal and ocular inflammatory reactions, which resolved over the follow-up period [66]. In a clinical study, it was shown that subretinal injection of the lentiviral vector was well tolerated, with no dose-limiting toxicities, and there was little or no ocular inflammation [67].

Baculoviruses (BV) are double-stranded DNA vectors, which are capable of transferring genes into various mammalian cells without viral replication [68]. Baculoviruses have high transgene capacity and have low cytotoxicity. It has been shown that baculovirus transduces different cells in the eye. Subretinal injection of baculovirus encoding GFP under the CMV promoter (rBV-CMV-GFP) led to GFP expression in RPE cells, which peaked 1–4 days after injection but was not detected after 2 months [69]. When rBV-CMV-GFP was injected intravitreally, GFP expression was detected in the corneal endothelium, the retinal inner nuclear layer, the ganglion cell layer, and the RPE cell layer. After 2 days of subretinal injection, the infiltration of macrophages was observed in the subretinal space and across the retina and after intravitreal injection in the anterior chamber and vitreous. In addition after 3 days, CD11b+ and CD4+ cells were found, but after 8 days, inflammation was almost completely resolved. In another study, intravitreally injected BV-GFP led to GFP expression in the photoreceptor cells and RPE after 6 days of injection [45]. In this study, inflammatory responses were detected in the anterior segment, retina, and the optic nerve head of the eye.

## 5. Preclinical Anti-Angiogenic Studies

### 5.1. VEGF-Blocking Agents

In preclinical in vivo studies, the most commonly used viral vector has been AAV during the last 3 years. Some studies have been conducted with adeno- and lentivirus vectors (Table 1). One way to inhibit VEGF is to use VEGF-blocking antibodies. In one study, choroidal neovascularization (CNV) was prevented by the inducible overexpression of aflibercept with a single injection of the AAV vector. Aflibercept inducibility was achieved by using riboswitches [70]. Riboswitches are gene control elements found in archaea, bacteria, and eukaryotes [71]. These gene control elements regulate gene expression by directly binding to a specific ligand. Most riboswitches are cis-acting gene control elements that reside usually in the 5′-untranslated region (UTR) that consists of an aptamer domain (ligand sensing structure) and an expression platform, which together undergoes conformational alterations upon ligand binding. A secretable aflibercept transgene cassette was delivered using an AAV capsid mutant vector with improved retinal penetrance [70]. TC45 riboswitch was incorporated in the 3’-UTR of the expression cassette and modulation of intraocular concentration of aflibercept was possible through the oral dosing of tetracycline as an activating ligand. Regular C57BL/6J mice were intravitreally injected with these constructs. After 7 weeks of injections and 7 days of CNV induction by laser, it was shown that constitutive expression of aflibercept prevented CNV formation significantly.

A codon-optimized cDNA of the aflibercept protein is encoded in AAV2.7m8 capsid, ADVM-022 [72]. It was shown that in non-human primates (African green monkeys), intravitreal injection of ADVM-022 suppressed exudative lesions and subretinal fibro-vascular complexes in CNV 2 and 4 weeks after the laser induction. The injection was made 13 months before laser induction and there were no clinical signs indicative of ADVM-022-related systemic effects observed.

A VEGF-blocking antibody, single-chain fragment variable (scFv) antibody, which lacks the Fc domain, was expressed via AAV2/8 with a strong CAG promoter and a WPRE (woodchuck hepatitis virus post-transcriptional regulatory element) sequence to enhance expression [73]. AAV-anti-VEGF-scFv was shown to reduce CNV in the laser-induced CNV mouse model.

RGX-314 is an AAV8 vector containing an expression cassette for a humanized antibody fragment that binds human VEGF (anti-VEGFfab) [27,51]. It was shown that subretinal injection of RGX-314 suppressed neovascular areas in transgenic mice expressing human VEGF in the retina. In a rat model of hVEGF, suprachoroidal injection of RGX-314 between the sclera and choroid suppressed VEGF-induced vascular leakage and vasodilation [27].

Pathological angiogenesis can be inhibited via soluble VEGF receptors [74]. In a study, intravitreally injected AAV2 expressing a soluble variant of VEGF receptor-1 decreased CNV lesion size 14 days after laser induction in the laser-induced CNV mouse model. Additionally, the infiltrated number of inflammatory and apoptotic cells was decreased.

### 5.2. Other Pro-Angiogenic Factors

Many other pro-angiogenic and anti-angiogenic factors have also been studied to inhibit angiogenesis. A mammalian target of rapamycin (mTOR) is a serine-threonine protein kinase [75]. The mTOR signaling pathway consists of two complexes: mTOR complex 1 and 2. mTOR1 is sensitive to rapamycin and responsible for cell growth, proliferation, angiogenesis, protein and lipid synthesis, and autophagy regulation. Recombinant AAV derived from self-complementary AAV2, expressing a short hairpin RNA, blocks the activity of mTOR complexes 1 and −2. Intravitreal injection of AAV-mTOR shRNA was shown to suppress angiogenesis and inflammation in a laser-induced CNV mouse model [75] and reduce neovascularization in a rat oxygen-induced retinopathy (OIR) model [76]. In another study, the optimal viral genome size for efficient AAV assembly was ensured by using a stuffer DNA derived from the 3’ UTR of the human UBE3A gene [77]. It was shown that this vector reduced CNV in the laser-induced mouse model.

Endostatin is an endogenous anti-angiogenic protein that downregulates HIF-1α [88]. Endostatin has been reported to reduce CNV [89]. In one study, an AAV2 vector comprising an RPE-specific promotor and HRE was used to regulate human endostatin production [78], and after subretinal injection, it reduced CNV areas 80% in a mouse model of laser-induced CNV after 21 days of injection. Endostatin mRNA levels were increased significantly at 3, 7, and 14 days after the laser and returned to baseline levels after 45 days.

Calreticulin anti-angiogenic domain (CAD) comprising amino acids 1–180 is a potent endogenous inhibitor of angiogenesis and has anti-inflammatory properties [90]. Intravitreal injection of AAV2 expressing CAD significantly suppressed retinal neovascularization in an OIR rat model [79]. Subretinal injection of this vector also reduced CNV lesions in the laser-induced mouse model after 21 days of subretinal injection and 14 days after laser induction.

Hepatocyte growth factor (HGF) is a mitogen that promotes epithelial and endothelial cell motility, cell migration, branching, and/or tubular morphogenesis and may induce VEGF production in different cells [91]. Kringle1 domain of HGF was shown to exhibit both anti-angiogenic and antitumor cell effects [92]. Intravitreal injection of AAV expressing the Kringle1 domain of HGF inhibited pathological retinal neovascularization in an OIR mouse model compared with vehicle [80]. The treated group showed good vascular distribution and the non-perfusion area was limited to the optic papilla, indicating that the overexpression of this fragment may be a potent treatment for ocular neovascularization.

Galectins are carbohydrate-binding proteins that are essential for tumor angiogenesis [93]. Galectin-1 (GAL-1) is linked to cell adhesion, migration, survival, and signaling and is associated with inflammatory disorders, cancer [93], and with proliferative diabetic retinopathy [94]. Adenoviral Gal-1-RNA interference was used to silence Gal-1 [81]. Intravitreal injection of d-Gal-1-RNAi decreased significantly retinal neovascularization and retinal hypoxia in the OIR mouse model.

### 5.3. Preclinical Studies with Genome Editing

Genome editing has become possible in living organisms [95]. In a single event, it is possible to add desirable or to remove undesirable alleles. A highly efficient way to edit the genome is the clustered regularly interspaced short palindromic repeats (CRISPR)/Cas9 (CRISPR associated protein) system. This system targets DNA sequences via nucleotide base pairing and RNA-guided endonuclease. There are different types of CRISPR/Cas systems. The AAV1-mediated CRISPR/Cas9 driven by an endothelial cell-specific promoter pICAM2 was used to deplete VEGFR-2 in vascular endothelial cells [82]. It was shown that AAV-CRISPR/Cas9 suppressed neovascularization in the mouse models of OIR and laser-induced CNV. Type V CRISPR/Cas system contains a large protein called Cpf1 [96]. Cpf1 differs from Cas9 in several ways. Cpf1 (Lachnospiraceae bacterium ND2006) was targeted to VEGF and HIF1α [83]. Intravitreally injected AAV9-Cpf1-Vegfa or -Hif1a reduced the area of CNV in a laser-induced CNV mouse model 1 week after laser induction and 6 weeks after injection. AAV-Cpf1-Vegfa or -Hif1a also reduced VEGF protein levels in RPE. There were no off-target effects in the retina or RPE cells.

### 5.4. Preclinical Studies with Multigenic Viral Vectors

The application of a multigenic viral vector producing multiple microRNAs has been used in anti-angiogenic studies. The phrase “multigenic” means that the vector can hold multiple gene cassettes for co-expression of several therapeutic factors. The combined expression of microRNAs targeting VEGF-A mRNA and fluorescent reporters was enabled by a lentiviral vector carrying back-to-back RNApolII-driven expression cassettes [62]. Expression of miRNAs was targeted to RPE cells by including a vitelliform macular dystrophy 2 VMD2 promotor. It was shown that subretinal injection of lentiviral anti-VEGF miRNAs reduced choroidal neovascularization in the laser-induced CNV mouse model 21 days after injection. Lentiviral-eGFP expression was shown to be stable and widespread 6 days, 21 days, and even 9 months after injection.

Multigenic AAV vectors expressing anti-VEGF miRNA and a secreted anti-angiogenic protein pigment endothelial-derived factor (PEDF) driven by the RPE-specific promoter provide improved protection against CNV [84]. It was shown that 57 days after subretinal injection of AAV5 vector either expressing anti-angiogenic miRNAs or co-expression of anti-angiogenic miRNAs and PEDF significantly decreased CNV area in a laser-induced CNV mouse model. In addition, EGFP expression was assessed after 57 days and it revealed the efficient transduction of RPE cells in both AAV5 expressing anti-angiogenic miRNAs but lower expression with co-expression of PEDF. PEDF is a member of the serine protease inhibitor family and acts as a multifunctional protein in different physiological and pathophysiological mechanisms such as angiogenesis, inflammatory responses, and neuroprotection [97]. PEDF functions as a major anti-angiogenic factor in the retina and vitreous body.

### 5.5. Preclinical Studies of Corneal Neovascularization

Not many studies to treat corneal neovascularization have been conducted. In one study, it was shown that intrastromal injection of an AAV vector containing codon-optimized human leucocyte antigen G1+ G5 (scAAV8G9- optHLA-G1 + G5) prevented corneal neovascularization and decreased the immune cell infiltration into the cornea over 2 months after the chemical corneal burn in rabbits [85]. However, one strong and two very mild scAAV8G9 capsid-specific neutralizing antibody responses were detected. HLA-G has immune-inhibitory functions and it is well known to protect the fetus from destruction by the mother’s immune system [98]. It also acts as an immune escape mechanism for tumors. HLA-G binds immune cells and inhibits their function. HLA-G protein was detected in adult human corneas, which means that it may contribute to the maintenance of the privileged immune status of the cornea [99].

### 5.6. Preclinical Studies of Retinopathy of Prematurity

VEGF inhibition is an attractive treatment for ROP, but it might delay or halt normal retinal vascularization and it is shown to have systemic implications such as lowering VEGF serum levels [100]. This raises concerns about the effects on developing organ systems, including the lungs and brain. Thus, other methods inhibiting neovascularization have to be considered. In one study, endothelial-cell-specific knockdown of VEGFR-2 or downstream STAT3 was shown to inhibit VEGF-induced retinopathy without delaying physiologic retinal vascular development [86]. Lentivirus vector under the VE-cadherin promoter was used to deliver shRNAs to knockdown VEGFR-2 or STAT3. The subretinal injection of these vectors reduced significantly intravitreal neovascularization in the rat OIR model that represents human ROP.

In another study, shRNA to VEGF was developed to inhibit the overexpression of VEGF-A in Müller cells [87]. It was shown that knockdown of a VEGF by a subretinal injection of lentivirus carrying shRNA driven by a cell-specific promoter significantly reduced intravitreal neovascular areas in the rat OIR model. Thinning of the photoreceptor and ONL layers was also detected, suggesting that knockdown of VEGF in Müller cells may have harmful effects on photoreceptors.

## 6. Gene Therapy Clinical Trials of Neovascular Ocular Diseases

In 2019, there were 37 gene therapy clinical trials for ocular diseases, constituting 1.2% of all gene therapy clinical trials (Table 2) [101]. In autumn 2020, there were seven active, recruiting clinical trials for ocular neovascularization utilizing three different gene therapy applications: soluble CD59, cDNA of aflibercept, and soluble anti-VEGF monoclonal antibody fragment related to ranibizumab. Thus, all the studies are based on AAV vectors and rely on a single administration. In addition, 4 out of 7 studies are in Phase I, assessing their efficacy and safety, whereas the rest are Phase II studies.

### 6.1. AAVCAGsCD59

Unlike the other ongoing clinical studies, the AAVCAGsCD59 gene therapy trial uses a novel target for treating neovascular ocular diseases. CD59 is a glycoprotein expressed in several cell types, such as endothelial cells [111]. Normal cells produce CD59 that blocks membrane attack complex (MAC), but the trend towards increased MAC expression [112] and reduced expression of CD59 is evident in both atrophic [113] and neovascular AMD patients [114]. Thus, as the complement cascade is upregulated, more MAC is formed than the body can protect itself against leading to cell damage and death.

AAVCAGsCD59 gene therapy (NCT03585556) utilizes AAV2 expressing soluble CD59 [102]. Gene therapy is intended to protect retinal cells that are responsible for central vision. The soluble recombinant version of the naturally occurring CD59 inhibits the formation of the MAC and prevents binding of the C9 proteins required to complete the terminal step of the complement-mediated cell lysis. In the Phase 1 study, the number of required anti-VEGF antibody injections after a single intravitreal injection of AAVCAGsCD59 is evaluated. Anti-VEGF is injected into patients with treatment of new onset wet AMD at day 0 to treat the CNV per standard of care and enable the AAVCAGsCD59 adequate time to enter the ganglion cells in the retina and start producing the transgene product. Seven days after anti-VEGF injection, AAVCAGsCD59 is administered at a dose of 3.6 × 10^11^ vg or 1.1 × 10^12^ vg via intravitreal injection. Patients will be followed for 12 months and treated with monthly anti-VEGF injections if neovascular AMD progresses. A long-term follow-up safety exam will occur 24 months post-injection.

### 6.2. ADVM-022

Two studies utilize the AAV vector for the expression of aflibercept (ADVM-022). The study subjects are assigned to receive one of the two doses (2 × 10^11^ vg/eye or 6 × 10^11^ vg/eye) of ADVM-022 intravitreally [103,115].

The Phase 1 study (NCT03748784) will evaluate subjects with active choroidal neovascularization secondary to AMD [103]. The primary endpoint for this study is safety and tolerability of ADVM-022, but key secondary outcome measures include the effect of ADVM-022 on the best corrected visual acuity, the effect of ADVM-022 on anatomic outcomes as measured by spectral-domain optical coherence tomography (SD-OCT), and the need for rescue aflibercept injections. All subjects will continue to be assessed for 104 weeks following the treatment with ADVM-022. In interim data, beyond 15 months from the gene transfer, no serious adverse events were noted, without the need for any rescue injections in the high-dose group [116].

Based on the promising results from the Phase 1 clinical trial of ADVM-022 in patients with wet AMD [104], a Phase 2 gene therapy trial for diabetic macular edema (DME) (NCT04418427) has been started to assess the durability of a single intravitreal injection of ADVM-022 [115]. Study subjects will be assigned to receive one of the two doses of ADVM-022 and further randomized to receive a preceding aflibercept or sham ocular injection. The control group will receive a sham ocular injection with a preceding aflibercept injection. All subjects will be assessed regularly and will receive additional aflibercept injections as needed. All subjects will be followed for 48 weeks after randomization.

### 6.3. RGX-314

The third study drug is RGX-314, an AAV8 vector containing a transgene for a soluble anti-VEGF monoclonal antibody fragment related to ranibizumab [105]. The expressed protein is designed to neutralize VEGF activity, modifying the pathway for the formation of new leaky blood vessels and retinal fluid accumulation.

The first Phase I/IIa dose-escalation study is for evaluating the safety and tolerability of RGX-314 gene therapy in subjects with previously treated neovascular AMD (NCT03066258) [105]. Five doses (3.9 × 10^9^ GC/eye to 2.5 × 10^11^ GC/eye) of subretinal delivery of RGX-314 will be studied. Safety will be the primary focus for the initial 24 weeks after RGX-314 administration. Following the completion of the primary study period, subjects will continue to be assessed until 104 weeks following the treatment with RGX-314.

There is also a long-term follow-up study of RGX-314 (NCT03999801) [106] for participants who were previously enrolled in the clinical study in which they received a single subretinal administration of RGX-314. This new study is a prospective, observational trial designed to evaluate the long-term safety of RGX-314 for up to 5 years post-RGX-314 administration, inclusive of the parent study.

The results are not yet published, but based on preliminary data from the sponsor, RGX-314 is well-tolerated across all cohorts, with no drug-related serious adverse events [117]. Sixteen serious adverse events not related to RGX-314, including two ocular procedure-related serious adverse events, were reported in ten patients. Positive long-term potential efficacy signals were sustained over 2 years in patients who received 6 × 10^10^ GC/eye. In this group, the mean change in visual acuity across all six patients was markedly improved over 2 years, with a mean best corrected visual acuity (BCVA) improvement of +14 letters.

In addition, patients in this group demonstrated long-term reductions in anti-VEGF treatment burden, and three out of six patients did not receive anti-VEGF injections over 2 years following a single administration of RGX-314. The mean annualized rate of anti-VEGF injections after gene therapy was 2.8 injections while one out of six patients received four anti-VEGF injections in the first 9 months.

The mean RGX-314 protein expression level in patients who did not receive anti-VEGF injections after 9 months was 273.6 ng/mL at 6 months and 291.7 ng/mL at 2 years compared to the mean protein expression levels of 217.8 ng/mL at 6 months and 227.2 ng/mL at 2 years in the group receiving a viral dose of 6 × 10^10^ GC/eye. Results show that long-term intraocular RGX-314 protein expression was stable in this group over 2 years. Consistent with previous results, intraocular RGX-314 protein expression levels increased up to a mean protein level of 848.7 ng/mL in a dose-dependent manner across cohorts.

A Phase 2 study utilizes suprachoroidal space as an administration route for RGX-314 gene therapy. A dose-escalation study evaluates the efficacy, safety, and tolerability of RGX-314 gene therapy in subjects with neovascular AMD (NCT04514653) [107] with doses of 2.5 × 10^11^ GC/eye or 5 × 10^11^ GC/eye [118]. Suprachoroidal gene therapy with RGX-314 is compared to intravitreal injection of monthly 0.5 mg ranibizumab. The suprachoroidal administration route is also exploited in a Phase 2 study for participants with nonproliferative and proliferative DR without center-involved diabetic macular edema (NCT04567550) [108].

### 6.4. Completed Clinical Trials for Ocular Neovascularization

The completed viral-vector-mediated gene therapy trials for ocular neovascular diseases from previous years have mostly relied on AAV as the gene carrier. The studies applied anti-angiogenic factors or soluble VEGF receptors to find new treatments for AMD.

### 6.5. sFLT-1

Soluble FLT-1 encoded by AAV2 has been utilized in several studies with neovascular AMD patients. sFLT-1 is a soluble human VEGFR-1 acting as a VEGF antagonist [119]. In the first Phase 1 study, gene therapy was reported to be safe and well-tolerated. All patients received ranibizumab injections at baseline and in week 4 [109]. A week after the first injection, the patients in the gene therapy group received subretinal injection of 100 μL of the AAV2 with a dose 1 × 10^10^ vg or 1 × 10^11^ vg. Patients in the control group did not receive any gene therapy treatment.

Both treatment groups were well-tolerated, and no ocular or systemic adverse events were attributed to the vector [109]. Adverse events related to the study procedures were minor and noted in three of six patients, including subconjunctival or subretinal hemorrhage and mild cell debris in the anterior vitreous. There were no major differences between the treatment groups in terms of CPT, BCVA, and the number of rescue treatments.

Patients received an injection only to one eye [109]. Nevertheless, vector shedding, both for vector DNA and viral capsid proteins, was detected in serum, urine, saliva, and tear samples from also the fellow eye. The rAAV.sFLT-1 vector DNA was detected by quantitative PCR only in tear specimens obtained 1 day after injection from the injected eyes of two patients but was undetectable in the next study point in 3 weeks. VEGF concentrations in the serum remained unchanged during the whole trial. At the end of the study at week 52, assessment in the rAAV.sFLT-1-treated patients showed no evidence of vision loss or atrophy in the paramacular area of the bleb site. In the gene therapy group, the median center point thickness CPT was 549 μm at baseline, decreasing to 311 μm at week 52. In addition, the BCVA improved from a median of 40 Early Treatment Diabetic Retinopathy Study (EDTRS) letters at baseline to 50 EDTRS letters. Four of six patients in the treatment group required zero rescue injections, while the other two required one rescue injection each.

In another Phase 1 clinical trial, a single injection of one of the four dose-ranging cohorts (2 × 10⁸ vg to 2 × 10^10^ vg) was injected into AMD patients and followed up for 52 weeks [110]. Compared with the first study, the injection was administered intravitreally and there were no control groups. Gene therapy seemed to be safe and well-tolerated at all doses. Although the maximum tolerated dose was not detected, the patients receiving the highest dose of viral vector experienced likely study-drug-related adverse effects (pyrexia and intraocular inflammation) and a decrease in BCVA, whereas in patients receiving lower doses, the BCVA increased during the study, whereas the mean change from baseline BCVA in patients receiving 2 × 10^10^ vg showed a slight early improvement followed by a gradual decline, but overall little change. In patients with intraretinal or subretinal fluid at baseline judged to be reversible, of those six showed fluid reduction, whereas five showed no fluid reduction.

No sFLT01 was detectable in aqueous humor in patients receiving lower doses, but five of ten patients who received an injection of 2 × 10^10^ vg had detectable amounts of sFLT01 that peaked at 26 weeks and slightly decreased afterward [110]. AAV2-sFLT01 vector DNA sequences were not detected in the blood, nasopharynx, urine, or semen of any patients at any time point. Neither were there any systemic adverse events likely to be related to gene therapy.

In a Phase 2a study, a larger group (*n* = 32) of AMD patients was treated subretinally with rAAV.sFLT-1 (1 × 10^11^ vg) [55]. The study protocol was the same as in the Phase 1 trial of Rakoczy et al. [109]. No serious ocular adverse events were reported in the gene therapy group [55]. The most common ocular AE in the rAAV.sFLT-1 group was intraocular hemorrhage. The majority of cases were mild in nature and visually insignificant, with no permanent sequelae. The two mild ocular adverse events that were considered possibly to be related to rAAV.sFLT-1 were eye and anterior chamber inflammation. No systemic safety issues were observed relative to the treatment.

AAV2 capsid was not detected at any time point. None of the patients had detectable vector DNA in their blood. However, rAAV.sFLT-1 DNA was detected transiently in the tears from the treated eye in a subset of patients (13/21), in the tears of the fellow eye in one patient, in the saliva in one patient, and in the urine in one patient. All these occurrences resolved in 4 weeks.

In the gene therapy group, the median change in BCVA was 1.0 ETDRS letters from baseline to week 52, whereas, in the control group, the median change in BCVA from baseline was −5.0 ETDRS letters. Almost half (12/21) of patients in the gene therapy group experienced maintenance or improvement of vision versus 4/11 control patients. The difference in median change in CPT between the gene therapy and control group was 95.0 μm. The median number of ranibizumab injections was 2.0 for the gene therapy group compared to 4.0 for the control group.

Follow-up combined Phase 1 and 2 trials up to 3 years [109]. Between 1 and 3 years, no serious adverse events were related to gene therapy. Even though BVCA improved from baseline and remained higher at 1, 2, and 3 years in the patients receiving sFLT-1, the difference was not statistically significant. In addition, the gene therapy did not show superiority in CPT or the number of required VEGF injections compared with the control group.

### 6.6. RetinoStat

Besides an AAV vector, also other viral vectors have been used as the carrier. A study with lentiviral equine infectious anemia virus (EIAV) vector expressing anti-angiogenic factors endostatin and angiostatin (RetinoStat) evaluated the safety of the treatment after a single subretinal injection [67]. Doses of 2.4 × 10^4^, 2.4 × 10^5^, or 8.0 × 10^5^ transduction units (TU) were injected into AMD patients with neovascularization. The study had no control groups.

Each of the doses was well-tolerated, without dose-limiting toxicities [67]. However, there was a macular hole that was the only procedure-related serious adverse event. RNA sequences were detected in the plasma of one subject on day 0 but not thereafter. A vector dose-related increase in aqueous humor levels of endostatin and angiostatin with high reproducibility among subjects was seen. Aqueous humor levels remained stable through the last measurement at week 48.

Long-term follow-up study beyond week 48 demonstrated that endostatin and angiostatin expression was maintained at the last measurement (2.5 years in eight subjects and over 4 years in two subjects). However, the treatment did not achieve significant beneficial effects in visual acuity or anatomical outcomes. Seven subjects out of 15 in the highest dose group received anti-VEGF injections due to persistent or increased disease activity. In contrast, only one injection was given to one patient in other groups.

### 6.7. PEDF

In addition, an adenoviral vector has been used for gene delivery in anti-angiogenic ocular clinical trials. This is a study of Ad5-mediated gene therapy encoding cDNA for human pigment epithelium-derived factor (PEDF) [49]. PEDF, originally extracted from cultured human fetal retinal pigment epithelial cells [120], is an anti-angiogenic factor. It exists naturally in the human eye, but its levels are decreased in diseases characterized by ocular neovascularization, such as DR [121,122] and AMD [123,124].

In a Phase I study, AdPEDF was delivered once via intravitreal injection into one eye with the worst visual acuity [49]. The study intended to study the safety of gene therapy in patients with advanced neovascular AMD. Eight dose levels of AdPEDF from 10^6^ to 10^9.5^ particle units (PU) were investigated until 12 months without the presence of a control group. Intravitreous injection of AdPEDF was generally well-tolerated at all doses and no drug-related serious adverse events were discovered. Seven patients (25%) had transient inflammation. Sputum and urine were negative for replicating adenovirus 3 weeks after gene delivery. Anti-adenoviral neutralizing antibody titers did not consistently rise after intravitreous injection of AdPEDF, but several patients showed a small increase, and one patient had a substantial increase at week 3 that returned to baseline at week 12. Patients treated with the highest dose, 10^8^–10^9.5^ PU, showed stabilization in CNV lesion size and mild improvement in visual acuity (around +2 EDTRS letters) up to 1 year after injection.

## 7. Discussion

As VEGF is the main factor in ocular neovascularization, it is one of the most important targets in anti-angiogenic treatment [2]. Because VEGF, VEGFR-1, and VEGFR-2 are constitutively expressed in the eye, there is a possibility that the pharmacologic inhibition of VEGF bioactivity may have adverse consequences. VEGF is necessary for the maintenance of RPE and choriocapillaris [125]. CATT and IVAN clinical trials showed that monthly anti-VEGF therapy increased the risk of RPE atrophy. It is thought that anti-VEGF therapy with bevacizumab for ROP delays and/or halts normal retinal vascularization. In one study, it was shown that low doses of bevacizumab had a good retinal structural outcome [126]. More future studies are underway to test lower doses of bevacizumab, which may enhance normal retinal vascularization while posing less systemic risk, regardless of the fact that extended follow-up is needed for infants [100]. In one study, it was shown that anti-VEGF therapy suppresses serum VEGF levels. The lowering serum VEGF levels raise concerns about the effects on the developing organs such as the brain and lungs. However, none of the controlled studies have shown worse neural developmental outcomes in patients 2 years of age after intravitreal anti-VEGF treatment. In addition, despite possible concerns with biological anti-VEGF treatments, intraocular anti-angiogenic gene therapy has not shown any effect on serum VEGF levels 52 weeks post-gene-transfer [55,127].

The use of proper model organisms helps in studying treatments for ocular human diseases [128]. Larger animal models, such as primates for ocular diseases, particularly retinal diseases, could be more suitable for these purposes because they have macula like humans. However, these animals are expensive, genetically difficult to manipulate, and ethical concerns have to be taken into account. Rodents, especially mice, have become the most widely used model of different human diseases and ocular diseases even though mice and human eye and retina differ. Out of the 18 preclinical studies discussed here, 12 utilized mice as the test animals. Rabbits, primates, and rats were used in a few studies. Mice are quite cheap and easy to manage and genetically manipulate. Gene transfer efficiency is usually inversely related to host size [129]. Generally, several applications and vector systems work in mice but obtaining equal treatment efficacy in larger animals and humans is more difficult due to the limited tissue diffusion of the gene transfer vectors and larger volumes of the transfected tissues. When targeting eyes with gene transfer, this can be overcome relatively easily as it is small in size and target cells in the eye are reachable. In preclinical animal studies and clinical trials, both subretinal and intravitreal administration routes were mostly used. The only exception is RGX-314 trials, which used injection into suprachoroidal space both in rodent studies [27] and in human trials [107,108].

The number of patients in the Phase 1 and 2 studies is low. In the ongoing clinical trials of gene therapy for ocular neovascularization, the estimated number of participants is between 25 and 42 people [102,105,106]. A small number of participants could lead more easily to variability between research subjects and carry a risk of failing to prove the effectiveness of the study treatment. In addition, the primary objective of the Phase 1 studies is to assess safety and tolerability. Thus, visual improvement is not the intended main outcome. In Phase 1 trials, the need to avoid patients with good visual potential who stand to benefit from standard care, leads to the situation in which participants have typically already severe vision impairment and they have failed to improve or maintain vision on existing standard treatments. As several clinical trials in AMD patients have shown a poor response or resistance to anti-VEGF treatment over time [130,131], it can be speculated whether these kinds of patients are able to demonstrate the efficacy of the gene therapy.

Despite years of clinical trials for ocular neovascular diseases, the studies have not yet proceeded to Phase 3 studies. However, in 2017, the FDA approved a subretinal delivery of AAV2-mediated voretigene neparvovec-rzyl (Luxturna^®^, Spark Therapeutics, Inc., Philadephia, PA, USA) for the treatment of inherited biallelic RPE65 mutation-associated retinal dystrophy [8]. Luxturna^®^(Spark Therapeutics, Inc., Philadephia, PA, USA) delivers a normal copy of the gene encoding the retinal pigment epithelial 65-kDa protein to retinal cells [132]. Although Luxturna^®^(Spark Therapeutics, Inc., Philadephia, PA, USA) is intended to treat a single gene mutation, the approval of the gene therapy proves that it is a useful tool for treating ocular diseases.

The CRISPR-Cas9 genetic editing technique is considered the next-generation gene therapy in general [133]. While gene editing therapies entered clinical trials in 2010, the first clinical trial showing the disease-modifying efficacy was demonstrated only in recent years. CRISPR-Cas genome editing applications are widely studied in ophthalmologic genome surgery as well [134]. Although the first clinical trial for eye disorders using the CRISPR strategy is for inherited Leber congenital amaurosis 10 [135], the applications with viral vectors have been used in preclinical trials for anti-angiogenic treatment [82,83].

Another trend in gene therapy is the use of AAV vectors. There have been setbacks in clinical trials due to pre-existing immunity to the viral capsids [133]. Nevertheless, this has not been a problem in ocular gene therapy due to the eye’s relative degree of immune privilege, at least in subretinal injections, although pre-existing immunity to the AAV is highly prevalent in humans [136]. A relatively large portion of humans carry circulating antibodies against the AAV capsid and these neutralizing antibodies may prevent AAV vector transduction and transgene expression.

Whereas none of the patients receiving smaller viral doses had an increase in anti-AAV2 antibodies, in the groups receiving higher viral doses, eight of 13 (62%) patients showed an increase after intravitreal injection, although in some cases, the increase was modest [110]. It is discussed whether the pre-existing serum antibodies against AAV2 have a negative effect on transgene expression as it was shown in some patients to correlate with a lack of protein expression. In another study using AAV2 delivered gene therapy, five of six patients had no changes in antibody titers after subretinal injection [127]. Thus, AAV2 delivery and transduction do not appear to be altered to the same degree by pre-existing humoral immunity when the vector is delivered subretinally. There are efforts to overcome this immune obstacle, such as engineering of modified AAV capsids, immunosuppression regimens, and methods for temporary clearing of antibodies from circulation [133]. Natural exposure to different AAV serotypes can result in the production of different IgG subclass antibodies [137]. The highest neutralizing factor prevalence was observed with AAV2 and AAV1. It is possible to use other AAV serotypes to overcome this problem.

Although neutralizing antibodies to AAV exist, it was shown that second administration of AAV2 vector expressing RPE65 gene into the subretinal space in individuals with inherited retinal dystrophy to the second eye after previous exposure to AAV2 was safe and effective [57]. As long-term gene expression is required to treat ocular neovascular diseases, AAV has been considered as a suitable choice for gene delivery. Re-administration of viral vectors has not been used in anti-angiogenic gene therapy trials treating the elderly and clinical trials have relied on a single administration. Even after adenoviral-delivered gene therapy, mild improvement in visual acuity up to one year after intravitreal injection was seen [49].

## 8. Conclusions

In conclusion, the preclinical animal studies have shown promising results with anti-angiogenic gene therapy with viral vectors. Different serotypes of viruses have been shown to transduce ocular tissues efficiently, and by modifying vector design, it can be further improved. Anti-angiogenic therapies have been shown to be safe and well-tolerated and reduce angiogenesis significantly. While preclinical studies succeeded, clinical trials have not proceeded as fast. Different animal models, gene delivery methods, viral vector designs, and timing of treatment must be studied further in order to succeed also in clinical trials.

To conclude, the clinical trials of intraocular gene therapy for neovascularization have shown the treatment to be safe, without severe adverse events or systemic effects. However, given the small number of participants, the advanced stage of the disease in patients enrolled, and the nature of the early phase trials, they remain unable to unequivocally confirm the existence of gene therapy efficacy. Thus, after proving their safety, larger clinical trials with randomized controlled groups are needed to prove the biological effects of the gene therapy treatments on ocular neovascularization.

## Figures and Tables

**Table 1:** Preclinical animal studies with viral-vector-mediated gene therapy for ocular neovascularization.

Animalmodel	Vector	Injection Site	Anti-Angiogenic Factor	Indication	Reference
Laser induced CNV in mouse	rAAV2/2-riboswitch	intravitreal	aflibercept	wet AMD	[70]
Laser induced CNV in non-human primate	AAV2.7m8	intravitreal	codon-optimised cDNA of the aflibercept protein (ADVM-022)	wet AMD	[72]
Laser induced CNV in mouse	AAV2/8	subretinal	anti-VEGF single-chain fragment variable (scFv) antibody	wet AMD	[73]
Transgenic mice expressing human VEGF in retina (rho/VEGF mice), rats expressing human VEGF in retina	AAV8	subretinal, suprachoroidal	humanized antibody fragment that binds human VEGF (anti-VEGFfab) (RGX-314)	wet AMD	[27,51]
Laser induced CNV in mouse	AAV2	intravitreal	soluble variant of VEGFR-1	wet AMD	[74]
Oxygen-induced retinopathy in rat, laser induced CNV in mouse	AAV2	intravitreal	mTOR shRNA,	wet AMD, ROP, DR	[75,76,77]
Laser induced CNV in mouse	scAAV2	subretinal	endostatin	wet AMD	[78]
Oxygen-induced retinopathy in rat, laser induced CNV in mouse	scAAV2	intravitreal, subretinal	calreticulin anti-angiogenic domain (CAD)	wet AMD, DR	[79]
Oxygen-induced retinopathy in mouse	rAAV	intravitreal	Kringle1 domain of hepatocyte growth factor (HGFK1)	wet AMD, DR	[80]
Oxygen-induced retinopathy in mouse	Adenovirus GV120	intravitreal	Galetin-1-RNAinterference	ROP	[81]
Laser induced CNV and oxygen-induced retinopathy in mouse,	AAV1-CRISPR/Cas9	intravitreal	CRISPR/Cas9 targeted to VEGFR 2	wet AMD, ROP	[82]
Laser induced CNV in mouse	AAV9-CRISPR-LbCpf1	intravitreal	CRISPR-LbCpf1 targeted to VEGF A and HIF1α	wet AMD	[83]
Laser induced CNV in mouse	Lentivirus	subretinal	microRNAs targeting VEGF-A	wet AMD	[62]
Laser induced CNV in mouse	AAV5	subretinal	PEDF, multiple miRNAs targeting the VEGFA gene	wet AMD	[84]
Corneal chemical burn in a rabbit	AAV8/9 chimeric capsid (8G9)	corneal intrastromal	codon optimized human leucocyte antigen G1+ G5	Corneal neovascularization	[85]
Oxygen-induced retinopathy in rat	Lentivirus	subretinal	shRNAs to VEGFR2 or STAT3	ROP	[86]
Oxygen-induced retinopathy in rat	Lentivirus	subretinal	VEGF-A or VEGF-A164 shRNA	ROP	[87]

AAV = adeno-associated virus, ADV = adenovirus, AMD = age-related macular degeneration, CNV = choroidal neovascularization, DR = diabetic retinopathy, LV = lentivirus, ROP = retinopathy of prematurity.

**Table 2:** Ongoing and finished clinical trials with viral-vector-mediated gene therapy for ocular neovascularization.

Clinical Trial	Vector	Injection Site	Anti-Angiogenic Factor	Indication	Reference
AAVCAGsCD59 (NCT03585556)	AAV2	intravitreal	soluble CD59	wet AMD	[102]
ADVM-022 (NCT03748784)	AA7	intravitreal	codon-optimized cDNA of the aflibercept protein	wet AMD	[103]
ADVM-022 (NCT04418427)	AA7	intravitreal	codon-optimized cDNA of the aflibercept protein	diabetic macular edema	[104]
RXG-314 (NCT03066258, NCT03999801)	AAV8	subretinal	soluble anti-VEGF monoclonal antibody fragment related to ranibizumab	wet AMD	[105,106]
RXG-314 (NCT04514653)	AAV8	subrachoroidal	soluble anti-VEGF monoclonal antibody fragment related to ranibizumab	wet AMD	[107]
RXG-314 (NCT04567550)	AAV8	subrachoroidal	soluble anti-VEGF monoclonal antibody fragment related to ranibizumab	diabetic retinopathy	[108]
Soluble FLT-1 (NCT01494805)	AAV2	subretinal	Soluble FLT-1	wet AMD	[109]
Soluble FLT-1 (NCT01494805)	AAV2	subretinal	Soluble FLT-1	wet AMD	[55]
Soluble FLT-1 (NCT01024998)	AAV2	intravitreal	Soluble FLT-1	wet AMD	[110]
RetinoStat (NCT01301443, NCT01678872)	lentivirus	subretinal	endostatin and angiostatin	wet AMD	[67]
AdPEDF (NCT00109499)	Adenovirus 5	intravitreal	cDNA for human pigment epithelium-derived factor (PEDF)	wet AMD	[49]

AAV = adeno-associated virus, ADV = adenovirus, AMD = age-related macular degeneration, DR = diabetic retinopathy, LV = lentivirus, ROP = retinopathy of prematurity.

## Data Availability

Data sharing not applicable.
